# Risk of reoperation within 12 months following osteosynthesis of a displaced femoral neck fracture is linked mainly to initial fracture displacement while risk of death may be linked to bone quality: a cohort study from Danish Fracture Database

**DOI:** 10.1080/17453674.2019.1698503

**Published:** 2019-12-05

**Authors:** Anne M Nyholm, Henrik Palm, Håkon Sandholdt, Anders Troelsen, Kirill Gromov, DFDB COLLABORATORS

**Affiliations:** a Clinical Orthopaedic Researc Hvidovre (CORH), Department of Orthopaedics, Copenhagen University Hospital Hvidovre, Copenhagen;; b Department of Orthopaedics, Holbaek Sygehus, Holbaek;; c Department of Orthopaedics, Copenhagen University Hospital Bispebjerg, Copenhagen;; d Clinical Research Centre, Copenhagen University Hospital Hvidovre, Copenhagen, Denmark

## Abstract

Background and purpose — Most guidelines use patient age as a primary decision factor when choosing between osteosynthesis or arthroplasty in displaced femoral neck fractures. We evaluate reoperation and death risk within 1 year after osteosynthesis, and estimate the influence of age, sex, degree of displacement, and bone quality.

Patients and methods — All surgeries for femoral neck fractures with parallel implants (2 or 3 screws or pins) performed between December 2011 and November 2015 were collected from the Danish Fracture Database. Radiographs were analyzed for initial displacement, quality of reduction, protrusion, and angulation of implants. The bone quality was estimated using the cortical thickness index (CTI). Garden I and II type fractures with posterior tilt < 20° were excluded.

Results — 654 patients with a mean age of 69 years were included. 59% were female. 54% were Garden II with posterior tilt > 20° or Garden III, and 46% were Garden IV. Only 38% were adequately reduced. 19% underwent reoperation and 18% died within 12 months. Female sex, surgical delay between 12 and 24 hours vs. < 12 hours, Garden IV type fracture, inadequate reduction, and protrusion of an implant were associated with statistically significant increased reoperation risk. No significant association between reoperation and age, CTI, or the initial angulation of implants was found. Notably, CTI was linked inversely with death risk.

Interpretation — Reoperation risk is linked mainly to primary displacement and reduction of the fracture, with no apparent effect of age or bone quality. Bone quality may be linked with risk of death.

The existing guidelines for treatment of displaced femoral neck fractures differ in their recommendations: most rely primarily or solely on the age of the patient, with osteosynthesis for patients younger than 65–75 years of age and arthroplasty for patients above this age, while a few simply advise arthroplasty for all displaced femoral neck fractures (Palm and Teixidor 2015). However, in addition to patient age, several other patient-related factors are known at the time of the surgery and may be useful to guide the treatment—but the influence of these factors on risk of reoperation is not well investigated.

We evaluated the risk of reoperation and death within 1 year following osteosynthesis of displaced femoral neck fractures, and estimated the influence of the age and sex of the patient, the degree of fracture displacement, and bone quality, in order to provide further evidence for nuancing the decision process and to improve outcome after a displaced femoral neck fracture.

## Patients and methods

**Figure 1.**
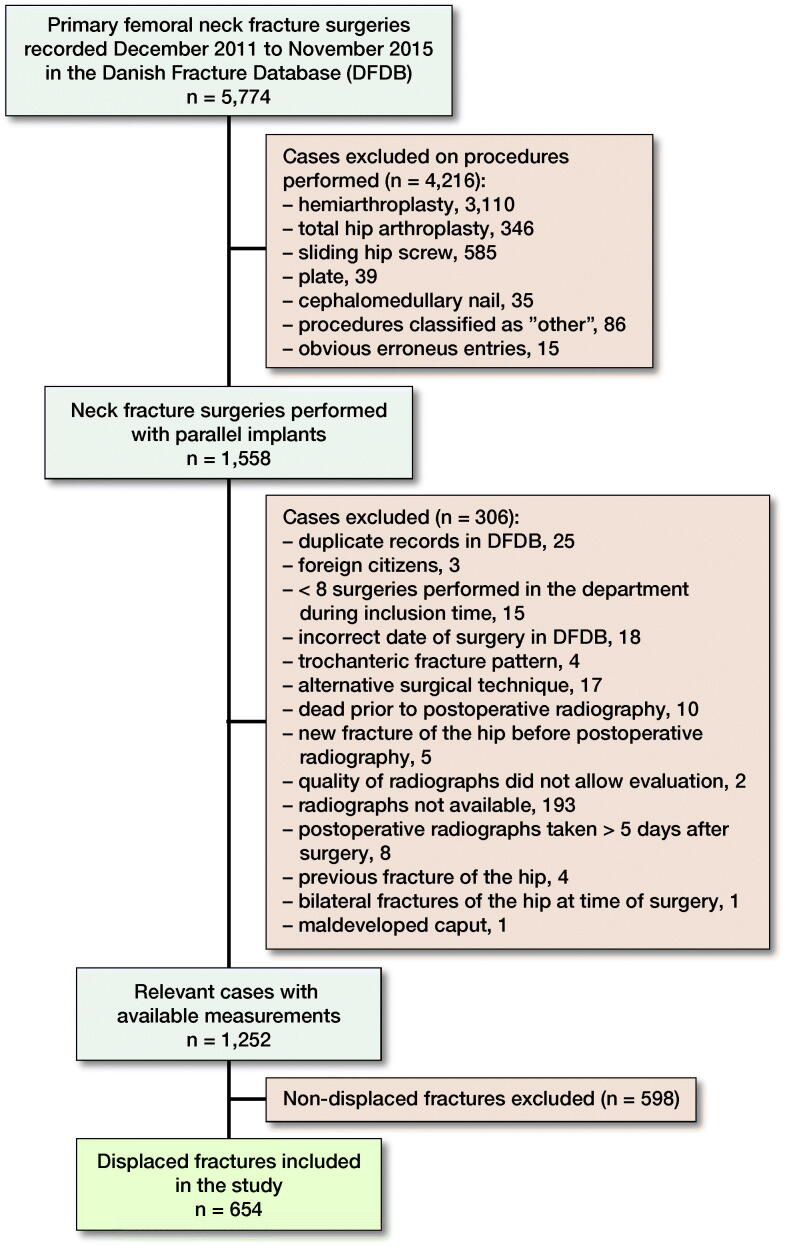
Case selection for inclusion in this study.

From December 2011 to November 2015, 5,774 surgeries for a primary femoral neck fracture (AO/OTA classification, 31B) were prospectively registered in the Danish Fracture Database (DFDB, www.dfdb.dk) (Gromov et al. 2014). Cases were selected for inclusion as described in a previous study of the same cohort (Nyholm et al. 2018), leaving 1,558 surgeries with use of screws or pins (parallel implants) (Figure 1). Data included age, sex, surgical delay, OTA/AO fracture classification, and ASA score. Time to surgery was defined as the time from fracture diagnosis (preoperative radiograph) until the onset of surgery.

**Figure 2.**
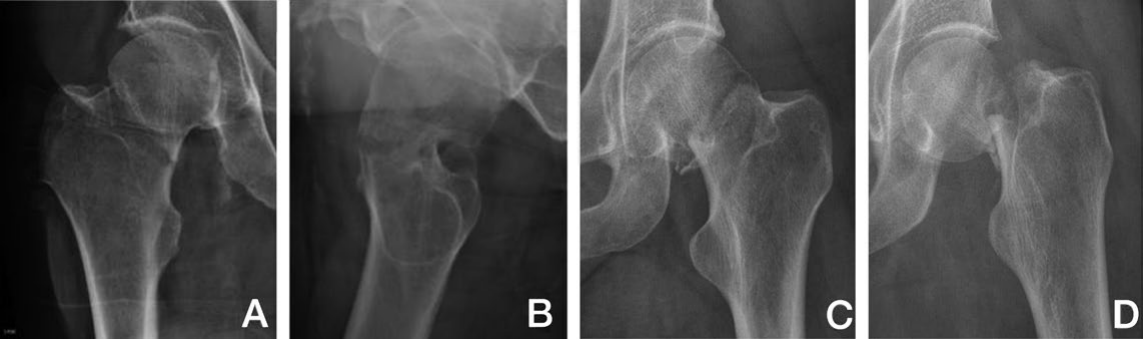
Degree of fracture displacement. The fractures were divided into 2 groups: “Mildly displaced” fractures: Garden type II fractures (A) with ≥ 20° posterior tilt measured on the axial view (B), Garden type III fractures (C), and “Severely displaced” fractures: Garden type IV fractures (D).

**Figure 3.**
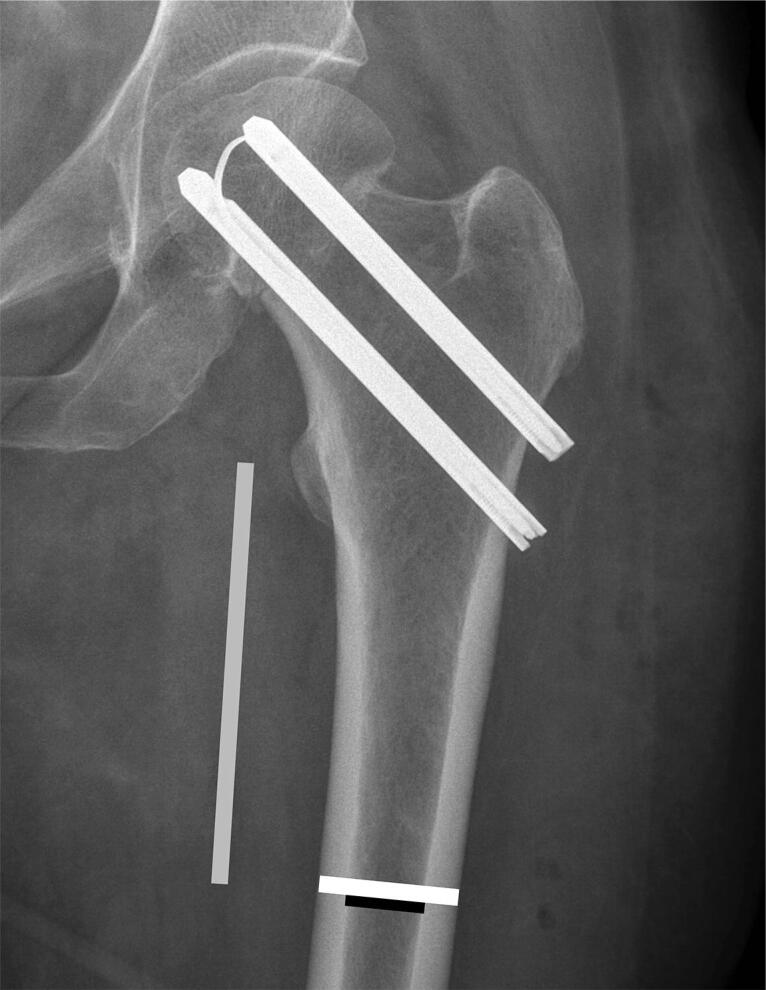
Cortical thickness index (CTI) is thickness of the cortices (white line minus black line) in relation to the diameter of the bone (white line) 10 cm below the tip of trochanter minor (grey vertical line). CTI = (white line – black line)/white line.

Pre- and postoperative radiographs (standard trauma AP and lateral view) of cases were collected from treating departments and analyzed for fracture displacement in accordance with the Garden classification (Figure 2), posterior tilt as measured by Palm et al. (2009), result of reduction (displacement and posterior tilt), implant protrusion into the joint (evaluated by eye), angle of implants to the lateral cortex of the femoral shaft measured as described by Nyholm et al. (2018) and cortical thickness index (CTI) measured as the part of the diameter of the femoral shaft that consisted of cortex measured 10 cm below the tip of the trochanter minor (Figure 3) as described by Sah et al. (2007). In this process 306 cases were excluded for various reasons (Figure 1), leaving 1,252 cases with available radiographs. Of these, 598 cases with initially non-displaced fractures with a posterior tilt of < 20° were excluded, leaving 654 cases with fracture types that according to guidelines are eligible for arthroplasty in patients ≥ 70 years of age (initially displaced fractures or non-displaced fractures with a posterior tilt ≥ 20°) for analysis (Figure 1) (Palm et al. 2012).

As described previously, intra- and inter-reader analyses were performed by 2 authors (HP and AMN), where measurements of 50 cases were performed twice with at least a 3-week interval between each read. This demonstrated a “Good” or Excellent” correlation for all included measures (Nyholm et al. 2018 and Table 1, see Supplementary data).

**Table 3.** Cox regression analysis of risk of reoperation

Factor	Univarible analyses HR (CI)	Multivariable analysis HR (CI)
Age		
Per 1 year increase	1.0 (0.99–1.01)	1.0 (0.99–1.02)
Sex		
Female	1	1
Male	0.64 (0.44–0.93)	0.57 (0.38–0.87)
ASA score		
1–2	1	1
3–4	1.3 (0.88–1.9)	1.5 (0.96–2.2)
Hours to surgery		
< 12	1	1
12–24	1.7 (1.1–2.6)	1.7 (1.1–2.6)
24–36	1.1 (0.53–2.1)	0.94 (0.44–2.0)
> 36	0.95 (0.42–2.2)	0.95 (0.40–2.3)
Test for overall effect	p = 0.05	p = 0.06
Cortical thickness index		
Per 0.1 increase	0.95 (0.76–1.2)	1.0 (0.80–1.4)
Fracture type		
Mildly displaced	1	1
Severely displaced	2.5 (1.8–3.7)	3.1 (2.0–4.7)
Reduction ^a^		
Fully reduced	1	1
Partly reduced	1.6 (0.92–2.9)	2.0 (1.1–3.8)
Not reduced	1.8 (1.2–2.7)	1.6 (1.1–2.5)
Test for overall effect	p < 0.01	p = 0.03
Angle of implants to the lateral cortex of the femoral shaft in AP		
> 125°	1	1
≤ 125°	2.0 (1.0–3.8)	1.7 (0.90–3.4)
Protrusion of implant		
No protrusion	1	1
Protrusion	2.1 (0.94–4.9)	2.4 (1.0–5.5)

**^a^**See footnotes Table 2.

Based on the radiographic measurements, the fractures were divided into 2 groups: “Mildly displaced” (Garden II type fractures with > 20° posterior tilt and Garden III type fractures) and “Severely displaced” (Garden IV type fracture) (see Figure 2). For the quality of the reduction, the fractures were divided into 3 groups: “Fully reduced” (non-displaced in AP view, < 10° posterior tilt), “Partly reduced” (non-displaced in AP view, > = 10° posterior tilt) and “Not reduced” (displaced in AP view).

After finishing radiographic analysis, data on any further surgery of the hip (ICD-10 KNF*) were collected from the National Patient Register (Landspatientregisteret, NPR) and analyzed to identify relevant reoperations as previously described (Nyholm et al. 2018). A relevant reoperation was defined as either a re-osteosynthesis of the primary fracture, an implant and femoral head removal, or an arthroplasty. Simple removal of the implants was not considered a relevant reoperation. Relevant reoperations were side-matched to the fracture surgery to ensure that the reoperation was not conducted in a contralateral hip. Data on vital status were collected from the NPR as well. Follow-up for all cases was 12 months.

### Statistics

The variables of interest were patient age, sex, initial fracture displacement, and bone quality (CTI). As surgical delay, result of reduction, protrusion of an implant into the joint, and angulation of the implants to the femoral shaft have previously been shown to influence risk of reoperation, these factors were all included as co-variables in an effort to optimize the models. The apparent effect of the included variables on the risk of reoperation was evaluated using Cox regression analysis. Time at risk was defined as time from the surgery until either reoperation, death, another non-relevant reoperation or surgery of the hip (reoperation for infection, a new fracture, femoral amputation), or end of follow-up.

Because death is a frequent occurrence in this population and influences the risk of reoperation in a patient, to support the interpretation of the analysis of risk of reoperation a separate Cox regression with death as outcome was performed. Time at risk was defined as time from surgery to death or end of follow-up.

For variables with several levels the overall effect in the model was evaluated using a likelihood ratio test. The fit of both models was evaluated using a proportional hazards test based on weighted residuals and was found to be acceptable. To evaluate the possibility of over-fitting, the variance of estimates in the model was compared with smaller models and found to be consistent, which suggest the models were not over-fitted.

To illustrate the magnitude of the risk of death and reoperation in different patient groups, several estimates of the probability of reoperation and death were made based on the Cox regression models for reoperation and death. 95% confidence intervals (CI) were used.

All data handling and analysis was performed using R software (version 3.4.3; 11/30/2017; R Foundation for Statistical Computing, Vienna, Austria) (R 2017).

### Ethics, registration, data sharing plan, funding, and potential conflicts of interests

This is a retrospective study, with all data collected from databases or radiographic analyses. No intervention was made, and the patients and families have not been contacted. There were therefore no ethical issues in relation to this study. A protocol with specified methods and outcomes was written prior to onset of the study. Permission to obtain and process data was obtained from the Danish Data Protection Agency (Datatilsynet, j.nr.: 2012-58-0004, local j.nr.: AHH-2015-032, I-Suite nr.: 03738) prior to the onset of the study. Study protocol and data managing/analysis files from R-studio will be available upon reasonable request; please contact corresponding author. Permissions to access data will have to be obtained from the relevant authorities, registries, and departments.

All costs were financed by the Department of Orthopaedics, Copenhagen University Hospital Hvidovre, Denmark. There were no conflicts of interest for any authors in relation to this study.

## Results

654 cases were included. Mean age was 69 years (21–102) and 385 (59%) were female. In 356 (54%) of the cases the fracture was mildly displaced (Garden II with posterior tilt > 20° or Garden III) and in 298 (46%) it was severely displaced (Garden IV) (Table 2). 28% had surgery within 12 hours, 78% within 24 hours, and 89% within 36 hours. 245 (38%) were adequately reduced, while the fracture was still ad latus displaced in the neck region on AP view or with > 10° posterior tilt in 409 (62%). In 18 (3%) cases an implant protruded into the joint. In 124 (19%) cases, the patient underwent a relevant reoperation, and in 117 (18%) cases the patient died. A larger proportion of the patients with a mildly displaced fracture died, but the patients in this group tended to be older (60% of patients with mild displacement were older than 70 years vs. 19% of patients with severely displaced fractures) (Table 2).

**Table 2.** Demographics and measurements of included cases. Values are frequency (%)

	Reoperated ^a^	Dead ^a^	Total ^b^
Factor	n = 124 (19) ^b^	n = 117 (18) ^b^	n = 654
Sex			
Male	40 (15)	53 (20)	269 (41)
Female	84 (22)	64 (17)	385 (59)
Age			
≤ 50	5 (9)	1 (2)	54 (8)
51–60	17 (16)	10 (9)	109 (17)
61–70	65 (29)	25 (11)	227 (35)
71–80	13 (13)	15 (15)	97 (15)
81–90	18 (17)	30 (29)	104 (16)
> 90	6 (10)	36 (58)	63 (10)
Cortical thickness index			
< 0.3	2 (13)	7 (46)	15 (2)
0.3–0.4	15 (19)	22 (28)	78 (12)
0.4–0.5	45 (19)	53 (22)	238 (36)
0.5–0.6	45 (17)	28 (11)	263 (40)
0.6–0.7	11 (25)	4 (9)	44 (7)
> 0.7	0 (	0 (	0 (0)
Hours to surgery			
< 12	27 (15)	25 (14)	182 (28)
12–24	75 (23)	61 (19)	325 (50)
24–36	12 (16)	13 (17)	77 (12)
36–48	5 (16)	7 (23)	31 (5)
> 48	2 (10)	7 (35)	20 (3)
Fracture displacement			
Mildly displaced ^c^	43 (12)	67 (19)	356 (54)
Severely displaced ^d^	81 (27)	50 (17)	298 (46)
Quality of reduction			
Fully reduced ^e^	33 (13)	38 (16)	245 (37)
Partly reduced ^f^	18 (21)	16 (19)	85 (13)
Not reduced ^g^	73 (23)	62 (19)	322 (49)
Angle of implants ^h^			
> 125°	112 (18)	111 (18)	621 (95)
≤ 125°	10 (33)	5 (17)	30 (5)
Protrusion of implant into the joint			
No protrusion	118 (19)	113 (18)	636 (97)
Protusion	6 (33)	4 (22)	18 (3)

**^a^**Percentage of the number of cases in each subgroup.

**^b^**Percentage of the total number of patients.

**^c^**Garden II with > 20° posterior tilt or Garden III type fracture.

**^d^**Garden IV type fracture.

**^e^**Non-displaced in AP view, < 10° posterior tilt (PT).

**^f^** Non-displaced in AP view, ≥ 10° PT.

**^g^**Displaced in AP view.

**^h^**Angle of implants to the lateral cortex of the femoral shaft in AP.

Female sex (HR 1.8; CI 1.2–2.6), surgical delay between 12 and 24 hours vs. < 12 hours (HR 1.7; CI 1.1–2.6), severe displacement (Garden IV type fracture, HR 3.1; CI 2.0–4.7), insufficient reduction (HR 1.7; CI 1.1–2.5), and protrusion of an implant HR 2.4 (CI 1.0–5.5) were associated with statistically significant increased risk of reoperation. No statistically significant association between reoperation and age, CTI, or the angulation of implants was found (Table 3).

In the death risk analysis increasing age of the patient, male sex, and high ASA score were associated with increasing risk of death. An inverse correlation between increasing CTI and risk of death was found (thinner cortex was associated with increased risk of death). Severely displaced fractures had a higher risk of death, but no statistically significant association with the quality of the reduction was found (Table 4).

**Table 4.** Cox regression analysis of risk of death

Factor	Univarible analysis HR (CI)	Multivariable analysis HR (CI)
Age		
Per 1 year increase	1.07 (1.05–1.08)	1.05 (1.03–1.07)
Sex		
Female	1	1
Male	1.1 (0.83–1.7)	1.6 (1.1–2.4)
ASA score		
1–2	1	1
3–4	5.7 (3.8–8.4)	3.7 (2.4–5.7)
Hours to surgery		
< 12	1	1
12–24	1.4 (0.88–2.4)	1.4 (0.85–2.2)
24–36	1.3 (0.64–2.5)	1.1 (0.58–2.3)
> 36	2.2 (1.1–4.1)	1.3 (0.65–2.6)
Test for overall effect	p = 0.2	p = 0.6
Cortical thickness index		
Per 0.1 increase	0.58 (0.48–0.71)	0.72 (0.58–0.89)
Fracture type		
Mildly displaced	1	1
Severely displaced	0.89 (0.62–1.3)	1.6 (1.1–2.5)
Reduction ^a^		
Fully reduced	1	1
Partly reduced	1.2 (0.68–2.2)	0.91 (0.48–1.7)
Not reduced	1.3 (0.84–1.9)	1.1 (0.69–1.7)
Test for overall effect	p = 0.5	p = 0.9
Angle of implants to the lateral cortex of the femoral shaft in AP		
> 125°	1	1
≤ 125°	0.94 (0.38–2.3)	0.85 (0.34–2.1)
Protrusion of implant		
No protrusion	1	1
Protrusion	1.3 (0.47–3.5)	2.0 (0.72–5.5)

**^a^**See footnotes Table 2.

Estimation of likelihood of death and reoperation for predefined patients with optimal surgical result (surgical delay < 12 hours, good reposition with implants angled > 125° to the lateral cortex of the femoral shaft, and no protrusion into the joint) demonstrated that risk of death depended in great part on the age, the sex, and the ASA score of the patient, while the risk of reoperation was primarily determined by the initial fracture displacement. A decrease in the CTI from 0.5 (average of the included group) to 0.4 (below the cut-off by Sah et al. (2007) for BMD-T score of –2.5) did not affect the risk of reoperation but did increase the risk of death for all estimates (Table 5).

**Table 5.** Estimates of risk of reoperation and death for predefined cases 1 year postoperatively. Values are percentages

Sex, ASA score		CTI 0.5		CTI 0.4	
Displacement		Estimated risk of		Estimated risk of	
	Age	death	reoperation	death	reoperation
Male, 1–2					
Mild	50	2	4	3	3
	60	4	4	5	4
	70	6	4	8	4
	80	10	4	14	4
Severe	50	3	10	5	10
	60	6	11	8	11
	70	10	12	13	12
	80	16	13	22	12
Male, 3–4					
Mild	50	8	5	11	5
	60	13	5	17	5
	70	21	6	28	6
	80	33	6	42	6
Severe	50	12	15	17	15
	60	20	16	27	16
	70	32	17	41	16
	80	48	18	59	17
Female, 1–2					
Mild	50	1	6	2	6
	60	2	6	3	6
	70	4	7	5	7
	80	7	7	9	7
Severe	50	2	17	3	17
	60	4	19	5	18
	70	6	20	9	19
	80	10	21	14	20
Female, 3–4					
Mild	50	5	9	7	9
	60	8	9	11	9
	70	14	10	18	10
	80	22	11	29	10
Severe	50	8	25	11	24
	60	13	26	18	26
	70	21	27	28	27
	80	33	29	43	28

All estimates are made with the surgical parameters as for an optimal surgery: surgical delay < 12 hours, reduction to non-displaced with < 10° posterior tilt, with implant-angle > 125° and without implant protrusion into the joint.

CTI = Cortical thickness index

For an 80-year-old female with a severely displaced fracture, the estimated risk of reoperation within 1 year is > 20% (Table 5). If, however, the fracture is only mildly displaced, the risk of reoperation for all patient types is < 10%, indicating that if no severely displaced fractures are treated with osteosynthesis with parallel implants, the risk of reoperation following osteosynthesis should be 3–10% (Table 5) (providing they are sufficiently reduced prior to fixation). In our cohort, 12% of the cases with mildly displaced fractures underwent a relevant reoperation (Table 2). If only cases with sufficiently reduced fractures were considered, the reoperation rate dropped to 8% (11 reoperations in 137 patients with only mildly displaced fractures that were sufficiently reduced).

## Discussion

In this registry-based study of risk factors for reoperation and death following displaced femoral neck fractures treated with osteosynthesis no significant association between patient age or cortical thickness index (CTI) and risk of reoperation was found. The main risk factors for reoperation were the amount of initial displacement, insufficient reduction, implant protrusion, increasing surgical delay, and female sex. In our secondary death risk analysis, an association between increased risk of death and increasing age, increasing ASA score, male sex, decreasing CTI, and severely displaced fracture type was found.

Although this study is based on consecutive patients with data collected prospectively in a nationwide database, the general limitations of observational studies still apply. The number of observations is limited and the fact that no statistically significant associations were found for several covariates may be due to lack of power in our sample and should be interpreted with care. A concern is that the patients in this study have been selected for osteosynthesis, as older patients with severely displaced fractures should primarily receive arthroplasty in accordance with Danish guidelines (Palm et al. 2012). The fact that we do not find any effect of age on risk of reoperation should therefore be interpreted with caution. The increase in risk of death with increasing age may well impact negatively on the risk of reoperation (patients who have died are not at risk of reoperation, and morbid patients may not receive a relevant reoperation due to poor health). The intra- and inter-reader measurements demonstrated a “good” or “excellent” correlation between the readers for all included measurements, indicating a reproducible reading of the radiographs, but the uncertainty between the 2D view seen on the radiographs and the 3D “reality” has not been validated and introduces an unknown uncertainty to the results. It is not our custom to follow these patients until healing and it was therefore not possible to evaluate the actual risk of non-union, avascular necrosis, or fracture displacement. Therefore, reoperation with secondary arthroplasty, revision of primary osteosynthesis, or femoral head removal was chosen as primary endpoint under the assumption that in our all-access, free-of-charge healthcare system all patients with clinically relevant complications such as pain and/or restriction of mobility would receive reoperation. It is, however, possible that some patients may not have undergone reoperation owing to patient-related causes. A follow-up of 12 months was chosen since previous studies with longer follow-up have demonstrated that 80–90% of all reoperations fall within this timeframe (Murphy et al. 2013), and the high mortality in this patient population is likely to introduce unnecessary confounding with a longer follow-up.

The risk factors for reoperation following femoral neck fractures have been evaluated in previous studies; however, most of those cohorts were quite small with less than 150 patients included. Our study, with 654 included cases, underlines the previous findings that for displaced femoral neck fractures a smaller initial displacement of the fracture in AP and/or lateral view (posterior tilt), as well as good reduction and avoiding protrusion of the implants into the hip joint, is associated with a reduced risk of subsequent reoperation (Bjørgul and Reikerås 2007, Hoelsbrekken et al. 2012, Yang et al. 2013). In contrast to the initial fracture displacement the latter 2 factors are both influenced by the surgeon and therefore possible to optimize. Several studies have demonstrated a better outcome with lower mortality as well as fewer healing complications and reoperations when the surgery is performed by a surgeon with experience in the specific procedure and performs it with some regularity (Strömqvist et al. 1992, Palm et al. 2007, Nyholm et al. 2015). Even though the procedure is generally viewed as less demanding, these findings underline the need for proper skill training and supervision of inexperienced surgeons as well as a potential benefit of concentrating the surgeries/supervision on fewer, but more experienced surgeons.

It has previously been suggested that poor bone quality is a major risk factor for failure following internal fixation of femoral neck fractures due to the association between poor bone quality and increased risk of primary fractures (Estrada et al. 2002). In our evaluation of the bone quality we chose to measure the bone quality by use of the CTI, which correlates well with BMD regardless of observer experience level (Nguyen et al. 2018) and is more easily accessible for the surgeon preoperatively than performing an acute gold standard DEXA scan (Sah et al. 2007, Nguyen et al. 2018). In contrast to this theory our study aligns with other newer studies in not finding such an association (Viberg et al. 2014). We did, however, find a quite strong inverse association between a low CTI and increased risk of death. Previous studies have demonstrated an association between poor bone quality and poor muscle quality (Papageorgiou et al. 2019) and it could thus be that the CTI is a surrogate measurement of the fitness and nutritional status of the patient. We have no information on the nutritional status of the included patients and therefore this is a theory to investigate in future studies. Based on the findings of our study, the CTI could be used as a marker to identify high-risk patients for postoperative mortality.

In line with our findings, risk of death has previously been associated with patient-related factors (age, sex, ASA score) and postoperative medical complications (Bjørgul and Reikerås 2007). Increasing surgical delay has previously been associated with an increasing risk of mortality following hip fracture (Khan et al. 2009, Nyholm et al. 2015), but the association with risk of reoperation has not been evaluated to the same extent. It has been suggested that expeditious treatment of displaced fractures is necessary to reduce the disturbance in blood supply for the femoral head and thus reduce the risk of avascular necrosis. In accordance with a previous study by Hoelsbrekken et al. (2012) we found that for initially displaced fractures increasing delay is associated with increased risk of later failure.

Whether to perform internal fixation or arthroplasty in displaced femoral neck fractures has been investigated quite extensively, primarily in patients older than 60–75 years of age (Parker and Gurusamy 2006, Rogmark and Johnell 2006) and, here, literature in general recommends a primary arthroplasty. The main argument is that studies with 12 months’ follow-up indicate lower risk of reoperation, less pain, faster re-convalescence, and better function, with no increased risk of mortality with arthroplasty (Gjertsen et al. 2010). Another often used argument for a primary arthroplasty in the elderly is the theory that risk of reoperation is increased with increasing age. As our study, in agreement with previous studies (Gregersen et al. 2015), did not support this theory, we feel this argument is weak. As a consequence, the argument for internal fixation in younger patient also weakens, which merits a lower age limit for when to insert an arthroplasty for a displaced femoral neck fracture. Although long term follow-up of primary arthroplasty in younger fracture patients is missing, arthroplasties for osteoarthrosis have in recent years achieved a 5-year and 20-year implant survival rate of 95% and 80% respectively (DHR 2016), and even among patients < 50 years it is 60–75% (DHR 2016). Furthermore, a larger number of younger hip fracture patients have been shown to be comorbid with either chronic diseases or disabilities and/or with an unhealthy lifestyle (tobacco and alcohol) (Rogmark et al. 2018) and these may therefore in many cases be regarded as fragility fractures in a population with a shorter life expectancy than a background population of the same age.

We therefore recommend re-thinking the indication for primary arthroplasty for displaced femoral neck fractures and basing the decision on whether patients are at risk of outliving an arthroplasty, thus needing reoperation later on. This would demand a broader evaluation of the patient’s risk factors for not only reoperation, but also of death, such as high ASA score, specific comorbidities, and perhaps also low CTI for optimizing the treatment of the individual patient. This merits routinely considering a primary prosthesis for fracture patients still of working age as a viable option, depending on the general medical fitness and activity level. The very youngest and fittest hip fracture patients have not been sufficiently evaluated in radiographic studies and, beyond theoretically superior fracture healing, these patients are at high risk of outliving their prosthesis due to both age and physical demands. In such patients much is to be gained from preserving their natural anatomy if at all possible, and in case of later fracture collapse and reoperation they are well suited for an elective secondary arthroplasty.

## Supplementary data

Table 1 is available as supplementary data in the online version of this article, http://dx.doi.org/10.1080/17453674.2019.1698503
