# Supplementary material for: Risk of reoperation within 12 months following osteosynthesis of a displaced femoral neck fracture is linked mainly to initial fracture displacement while risk of death may be linked to bone quality: a cohort study from Danish Fracture Database
Source: Acta Orthop. 2019 Dec 5;91(1):1–75. doi: 10.1080/17453674.2019.1698503 (PMC7006706; doi:10.1080/17453674.2019.1698503)
Supplement: Supplemental Material [file IORT_A_1698503_SM0780.pdf]

Supplementary data

Table 1. Intra- and interreader measurements

		CCC (CI) [a]	md [b]	SD (md)	CI (md)	LOA [c]	COV (%) [d]
Intraobserver Reader 1 (AMN)	Cortical index	0.92 (0.87–0.96)	0.00052	0.030	(–0.059 to 0.060)	(–0.060 to 0.059)	5.74
Intraobserver Reader 2 (HP)	Cortical index	0.93 (0.87–0.96)	0.0038	0.027	(–0.0037 to 0.011)	(–0.057 to 0.049)	5.32
Interobserver Reader 1 vs 2	Cortical index	0.90 (0.85–0.93)	0.013	0.030	(0.0066 to 0.019)	(–0.046 to 0.072)	5.82

[a] CCC = Lin's concordance correlation coefficient
[b] md = mean difference
[c] LOA = Limits of agreement
[d] COV = Coefficient of variation

References

[a] Lin L I. A concordance correlation coefficient to evaluate reproducibility. Biometrics 1989; 45(1): 255-68.
[c] Bland J M, Altman D G. Statistical methods for assessing agreement between two methods of clinical measurement. Lancet 1986; 327: 307-10. doi:10.1016/S0140-6736(86)90837-8
[d] Jones R. Clinical Investigation and statistics in laboratory medicine. London: ACB Venture Publications; 1997.
[d] Synek V. Evaluation of the standard deviation from duplicate results. Accredit Qual Assur 2008; 13(6): 335-7. doi:10.1007/s00769-008-0390-x
